# Penicillin Binding Proteins as Danger Signals: Meningococcal Penicillin Binding Protein 2 Activates Dendritic Cells through Toll-Like Receptor 4

**DOI:** 10.1371/journal.pone.0023995

**Published:** 2011-10-27

**Authors:** Marcelo Hill, Ala-Eddine Deghmane, Mercedes Segovia, Maria Leticia Zarantonelli, Gaëlle Tilly, Philippe Blancou, Gaëlle Bériou, Régis Josien, Ignacio Anegon, Eva Hong, Corinne Ruckly, Aude Antignac, Meriem El Ghachi, Ivo Gomperts Boneca, Muhamed-Kheir Taha, Maria Cristina Cuturi

**Affiliations:** 1 INSERM U643, Nantes, CHU de Nantes, IUN, Nantes, Université de Nantes, UMR 643, Nantes, France; 2 Departamento de Inmunobiologia, Facultad de Medicina, Universidad de la Republica, Montevideo, Uruguay; 3 Institut Pasteur, Invasive Bacterial Infections Unit, Paris, France; 4 Institut National de la Recherche Agronomique, Ecole Nationale Vétérinaire, Université de Nantes, Nantes, France; 5 Institut Pasteur, Biology and Genetic of Bacterial Cell Wall Group, Paris, France; 6 INSERM, Biology and Genetic of Bacterial Cell Wall Group Avenir, Paris, France; 7 CHU de Nantes, Laboratoire d'Immunologie, Nantes, France; Institute of Microbial Technology, India

## Abstract

*Neisseria meningitidis* is a human pathogen responsible for life-threatening inflammatory diseases. Meningococcal penicillin-binding proteins (PBPs) and particularly PBP2 are involved in bacterial resistance to β-lactams. Here we describe a novel function for PBP2 that activates human and mouse dendritic cells (DC) in a time and dose-dependent manner. PBP2 induces MHC II (LOGEC50 = 4.7 µg/ml±0.1), CD80 (LOGEC50 = 4.88 µg/ml±0.15) and CD86 (LOGEC50 = 5.36 µg/ml±0.1). This effect was abolished when DCs were co-treated with anti-PBP2 antibodies. PBP2-treated DCs displayed enhanced immunogenic properties *in vitro* and *in vivo*. Furthermore, proteins co-purified with PBP2 showed no effect on DC maturation. We show through different *in vivo* and *in vitro* approaches that this effect is not due to endotoxin contamination. At the mechanistic level, PBP2 induces nuclear localization of p65 NF-kB of 70.7±5.1% cells versus 12±2.6% in untreated DCs and needs TLR4 expression to mature DCs. Immunoprecipitation and blocking experiments showed that

PBP2 binds TLR4. In conclusion, we describe a novel function of meningococcal PBP2 as a pathogen associated molecular pattern (PAMP) at the host-pathogen interface that could be recognized by the immune system as a danger signal, promoting the development of immune responses.

## Introduction

The penicillin-binding proteins (PBPs) are conserved proteins which play a critical role in building the cell wall in several bacterial pathogens by catalyzing the biosynthesis of peptidoglycan [1]. Indeed, inhibition of PBPs produces an imbalance in cell wall metabolism resulting in growth arrest or lysis. β-lactam antibiotics covalently link PBPs and therefore act as suicide inhibitors of PBPs. Acquisition of PBPs with low affinity for the β-lactams is a mean of antibiotic resistance, in addition to a decreased permeability of the outer membrane, antibiotic export, or degradation by β-lactamases [2].

*Neisseria meningitidis*, a Gram-negative human pathogenic bacterium, infects asymptomatically the nasopharynx of about 10% of the population worldwide [3]. Occasionally, it provokes invasive infections leading to inflammatory diseases such as septicaemia, meningitis and arthritis. *N. meningitidis* contains three defined PBPs [4], [5]. PBP1 encoded by *ponA*, PBP2 encoded by *penA* and PBP3 encoded by *pbp3* [6]. Besides its role in peptidoglycan synthesis and penicillin G resistance, we have shown that PBP2 from *N. meningitidis* displays immunogenic properties. Indeed, sera from patients convalescent of meningococcal disease recognized PBP2s from different strains [7]. Moreover, vaccination with purified recombinant PBP2 and administration of purified anti-PBP2 rabbit IgG antibodies conferred protection against experimental meningococcemia in mice. Thus, PBP2 can be the target of a protective adaptive immune response [7].

We speculated that PBP2 from *N. meningitidis* could also constitute a pathogen-associated molecular pattern (PAMP) acting as a pro-inflammatory molecule on dendritic cells (DCs). DCs reside within the epithelium and represent a first line of defence against invading *N. meningitidis* [4]. Here we show for the first time that, in addition to the functions described above, PBP2 can also trigger DC maturation in a TLR4-dependant manner and therefore increase the immunogenic properties of DCs *in vitro* and *in vivo*. A novel role for meningococcal PBP2 as a PAMP is therefore described at the host-pathogen interface.

## Materials and Methods

### Ethics statement

All animal experiments were performed under specific pathogen-free conditions in accordance with the European Union Guidelines. All animal studies were conducted according to the guidelines of the French Agriculture Ministry. The studies were approved by the Veterinary Departmental Services committee, La Chapelle-Sur-Erdre, and Paris France (No:E.44011; No 75-1554), and all experiments were carried out in compliance with the ethical rules of the INSERM and the Institut Pasteur.

### Mice

The Ins-HA-transgenic mice [5] express HA of the influenza virus in pancreatic islets and the TCR-HA_512–520_ transgenic mice [7] express a TCR-specific for the H-2K^d^-restricted (IYSTVASSL) epitope of HA (Département de Cryopréservation, Typage, et Archivage Animal Orléans, France). TLR2^−/−^, TLR3^−/−^, TLR4^−/−^ mice (C57/BL6 H-2K^b^ background) were elevated at the Institut Pasteur (Paris, France). BALB/c mice were obtained from Janvier (France).

### Recombinant PBP2 production and purification

The recombinant plasmids pAA2 and pAD4 (pET28b harbouring *penA* or *crgA*, respectively) were described previously [6] *ponA* which encodes for meningococcal PBP1 lacking the signal peptide and the transmembrane domain (the first 30 codons) has been amplified by PCR from the strain 8013 using the oligonucleotides AA-16 (5′-GCTGGTCTCCCATGACGTATCCGAAACTGC-3′) and AA-17,(5′-CAGGCGGCCGCAAACAGGGAATCCAACTGC-3′) harbouring respectively *Bsa*I and *Not*I as adapters and then cloned into pET28b cut with NcoI and XhoI restriction enzymes. The gene *pbp2* of *Helicobacter pylori* (*Hp*) without the signal peptide and the transmembrane domain regions, was amplified from the strain 26695 [8], using the oligonucleotides pbp2-BamHI-pACYCduet (5′-CGCGGATCCGTTGGCTGAACGAAACATG-3′) and pbp2-NotI-pACYCduet (5′-AAGGAAAAAAGCGGCCGCTTAAAGATAGCCAAGCTCATAGAG-3′). The PCR product was digested with BamHI and NotI and inserted into pACYCduet (Novagen), which was cut with the same endonucleases. The recombinant plasmids were transformed into *E. coli* BL21(DE3) pLysS strain and His6-tag-recombinant proteins were overexpressed and purified using a nickel nitrilotriacetic acid-agarose column (Qiagen, Düren-Germany), as previously reported [6]. His6-tagged PBP2 was further purified using an anion exchange column (Mono Q HR 10/10, GE Healthcare). PBP2 was applied on to the column equilibrated with buffer A (20 mM Tris- HCl, pH 8; 150 mM NaCl). PBP2 was eluted using a linear NaCl gradient (from 0 M to 1.35 M), Protein concentrations were determined spectrophotometrically by monitoring the absorbance at 278 nm. The purity of PBP2 was confirmed by SDS-PAGE and silver staining as previously described [9].

### Endotoxin detection assay

The level of endotoxin in the purified preparations was determined by a quantitative, chromogenic QCL-1000 Limulus amoebocyte lysate (LAL) assay (Cambrex BioScience Walkersville, Inc., alkersville, MD, USA) according to the manufacturer recommendations. The detection limit of the assay was 0.01 EU/ml.

### Cell preparation, culture, and treatments

Bone marrow cells were cultured in RPMI 1640 medium supplemented with 10 ng/ml of supernatant from COS cells transfected with murine GM-CSF cDNA, 10% FCS, 2 mM L-glutamine, 100 U/ml penicillin, 0.1 mg/ml streptomycin, and 50 mM 2-ME (all from Sigma- Aldrich). At day 8, non-adherent cells were harvested and used for the different experiments. PBP2 was used at 10 µg/ml unless otherwise stated for 48 h. LPS (*E. coli* 0111:B4) was from Sigma-Aldrich and used at 50 ng/ml for 48 hs unless otherwise stated. Polymixine B (PMB, Sigma- Aldrich) was used at 10 µg/ml and incubated with LPS or PBP2 30 minutes before incubation with cells. For blocking experiments, PBP2 or LPS were pre-incubated with 5 µg polyclonal rabbit anti-PBP2 IgG [6] or irrelevant polyclonal antibody at 37°C for 1 h. In most experiments, BMDCs were generated using C57/BL6 mice. In diabetes induction experiments BMDCs were generated using BALB/c mice. DCs from mouse spleens were purified according to [10]. Human monocyte-derived DCs were generated as previously described [11].

### ELISA

Mouse ELISA kits (BD Pharmingen) for IL-12p70 and TNF-α were used to quantify these cytokines in the culture supernatant of treated DCs. Capsule polysaccharide were purified according to a modification of the previously reported method [12]. Cetavlon extraction (10%) was conducted at 4°C and followed by DNase and RNase treatment. After phenol extraction, Nm X capsular polysaccharide was recovered by ultracentrifugation, dialyzed and lyophilized (yield between 2 mg and 5 mg). Coating of purified PBP2 or purified capsular polysaccharide was performed in ELISA plates using suspension at 1 µg/ml concentration of protein or polysaccharide.

### Flow cytometry

BMDCs were stained with anti-CD40, MHC class II, CD80, CD86 and CD11c antibodies (BD Biosciences and Miltenyi Biotech). The staining was analyzed in the CD11c+ gate. We used a FACSCalibur flow cytometer and CellQuest software or a LSR II flow cytometer and FACSDiva software (BD Biosciences).

### MLR

C57/BL6 BMDCs were cultured at different ratios with 10^5^ BALB/c lymph node cells. Proliferation was studied at day three by analyzing DDAO-SE dilution by flow cytometry as previously described [13]. For human MLR, monocyte-derived DCs treated with PBP2 or not were cultured at different ratios with 5×10^4^ allogeneic purified T cells. Proliferation was assessed at day three by analyzing ^3^H-thymidine incorporation as described by [14].

### Diabetes induction

Six- to 8-wk-old Ins-HA mice were injected i.v. with 0.5×10^6^ CD8+ T cells (purity >95%) isolated from HA512–520 TCR-transgenic mice [5] (Miltenyi Biotec). Twenty-four hours later, mice were injected i.v. with 15,000 HA-loaded LPS-matured or PBP2-treated DCs. Diabetes was monitored using Clinistix urinalysis strips (Bayer). Mice were considered diabetic when the glucose concentration was above 5.5 mmol/L.

### Immunocytology

NF-κB nuclear translocation was analyzed by immunofluorescence and images were analyzed by confocal microscopy. An anti-p65 (F-6) monoclonal antibody from Santa Cruz Biotechnology (Santa Cruz, CA) was used at 5 µg/mL. The secondary antibody was an antimouse-Alexa568 from Jackson (Suffolk, UK). Cell nuclei were counterstained with TOPRO-3iodide (Molecular Probes, Eugene, OR) and slides were mounted in ProLong AntiFade reagent (Molecular Probes). Slides were analyzed with a Leica confocal microscope and the Leica TCS NT software.

#### Pull-down assays

Hec-1-B epithelial cells were treated with 10 µg of purified His6-tagged proteins. Cells were then lysed in extraction buffer (25 mM Tris-HCl pH, 7.5, 1 mM EDTA, 1 mM EGTA, 100 mM NaCl, 1% Triton X-100, 0.5% NP-40, 0.2 mM PMSF and protease inhibitor cocktail) for 20 minutes at 4°C and debris were removed by high speed centrifugation. Five hundred µg of soluble proteins were mixed with 5 µg of anti-hTLR4 mAb (clone 1G11, Sigma, France), or an irrelevant normal mouse IgGs. Alternatively, soluble proteins were mixed with 5 µg of anti- HisTag rabbit polyclonal antibody (Abcam, France) or an irrelevant normal rabbit IgGs. The mixture was incubated for 1 hour at 4°C. Protein A-agarose beads were added to the mixtures and the samples were incubated for an additional 30 minutes at 4°C. Agarose beads were washed extensively and protein complexes were solubilised in 1× Laemmeli buffer and submitted to SDS-PAGE and immunoblot using either anti-His Tag polyclonal antibody (for anti-TLR4 immunoprecipitated samples) or anti-TLR4 mAb (for anti-His-tag immunoprecipitated samples). Cells or purified proteins were used as controls as indicated.

#### Binding and blocking assay for PBP2/human TLR4

Hec-1-B cells were seeded in RPMI medium supplemented with 5% FBS in 24 well plate at a density of 10^5^ cells/well. Cells were then treated for 1 h at 37°C with increasing amounts of purified His-tagged PBP2 in presence of anti-TLR4 blocking antibodies (clone HTA125 eBioscience, Hatfield, UK) or an irrelevant normal mouse IgG (10 µg). After extensive wash with HBSS supplemented with 1% FBS, cells were fixed for 15 min with 3% paraformaldehyde. Bound PBP2 was detected using anti-His-tag polyclonal antibody followed by HRPO-conjugated goat anti-rabbit antibody. Colorimetric signal was detected using HRPO substrate and quantified using multiskan Ascent reader plate (Thermolab Systems) at 492 nm.

### Statistical analysis

Statistical significance was assessed using the nonparametric one-way ANOVA test with a Tukey post test. Differences were considered significant when *p*<0.05.

## Results

### PBP2 induces human and mouse phenotypic DC maturation

**Figure 1:**
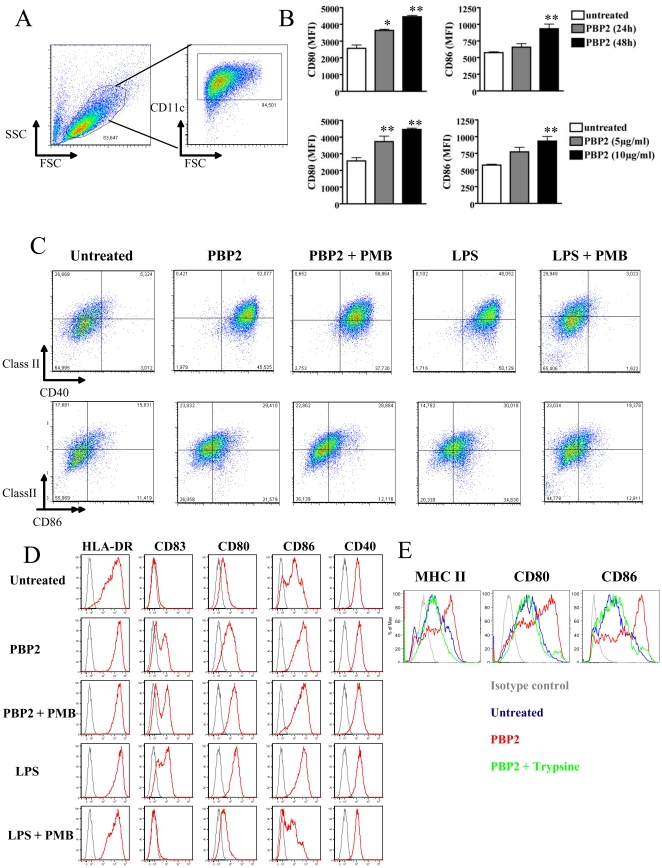
PBP2 induces DC maturation. **A.** Gating strategy used in all the experiments using mouse BMDCs. Different staining were then analyzed in the CD11c^+^ fraction. **B:** Mouse BMDCs were left untreated or cultured in the presence of 10 µg/ml PBP2 for 24 or 48 hours (upper panel) or with the indicated PBP2 doses for 48 hs (lower panel). CD80 and CD86 expressions were analyzed by FACS. Mean ± SD from three independent experiments is depicted in the graphs. *: p<0.05; **: p<0.01. **C:** PBP-2-induced phenotypic maturation of mouse BMDCs was analyzed by FACS after a 48 h culture in presence of PBP2 or LPS (from *E. coli* 0111:B4) alone or in combination with PMB. PMB did not affect PBP2-induced phenotypic maturation whereas it completely abolished LPS- induced expression of CD40, class II molecules and CD86. Numbers in the graphics represent the percentage of double-positive cells. n = 4. **D:** Human monocyte-derived DCs were treated for 48 h as for C. The expression of the indicated molecules was studied by FACS (red histogram plots). Isotype control staining is depicted in each graphic as grey histogram plots. Cell debris were excluded from the analysis by gating on live cells in the FSC/SSC plot. Representative results from 1 out of 4 donors are depicted. **E:** PBP2 was left untreated or treated for 1 h at 37°C with trypsin. The enzyme was then blocked with foetal calf serum and preparations were used to treat DCs for 48 hs. One experiment representative of three is shown.

The main objective of the present study was to determine whether PBP2 could be recognized by DCs as a danger signal and therefore increase its immunogenic properties. We therefore analyzed DC phenotypic maturation upon PBP2 treatment. We observed that PBP2 induced expression of CD80, CD86, CD40 and MHC class II on mouse bone marrow-derived DC (BMDC) (**Figure 1A–C**) in a dose and time-dependent manner (**Figure 1B** **and Figure S1**). Importantly, PBP2 also increased the expression of CD40, CD80, CD86, HLA-DR and the maturation marker CD83 in human monocyte-derived DCs (**Figure 1D**). Although endotoxin-free reagents were used during the purification process, it was critical to control for potential endotoxin contamination in the recombinant PBP2 preparations. PBP2 preparations were directly evaluated for the presence of bacterial endotoxin using the highly sensitive Limulus Amebocyte Lysate (LAL) assay (CAMBREX). The detection limit of the assay was 0.01 EU/ml. Maximum detectable endotoxin was at 0.314 EU/ml in a 10 µg/ml PBP2 sample. The minimum LPS concentration required to induce DC maturation under our experimental conditions in the present study was 1 EU/ml, three times greater than the maximal amount of LPS detectable in PBP2. To further address an eventual role of LPS contamination, we performed two different approaches. First, DCs were stimulated with PBP2 in the presence of the endotoxin-neutralizing antibiotic polymyxin B (PMB) and DC phenotype was assessed as described above. As expected, LPS-induced phenotype was completely abolished with PMB. In clear contrast, PBP2-induced mature phenotype was not affected by PMB treatment in mouse and human DCs (**Figure 1C–D**). Secondly, we observed that PBP2 degradation using trypsin completely abolished its effect on DC phenotype (**Figure 1E**). Taken together, we showed that PBP2 induces human and mouse DC maturation and that this effect is not due to endotoxin contamination. Most experiments were performed using affinity chromatography purified PBP2 as stated in MATERIALS AND METHODS. To exclude the possibility that the maturation of DC was the result of a potential co-purified contaminating protein, affinity chromatography issued PBP2 was further purified using anion-exchange chromatography (**Figure S2A and B**). Importantly, similar results (i.e. PBP2-induced DC maturation) were obtained using highly pure PBP2-containing fraction (F2), whereas other minor fractions lacking PBP2 (F1 and F3) did not induce DC maturation (**Figure S2C**). Furthermore, anti-PBP2 polyclonal antibodies completely abolished the effect of PBP2 preparations (**Figure S2D**). It is worth noting that altered PBP2 associated with decreased susceptibility to penicillin G was undistinguishable to “wild type” PBP2 in inducing DC phenotypic maturation (not shown). PBP2 treatment did not affect DC viability as assessed through propidium iodide staining (not shown). These data unambiguously showed that PBP2 induces DC maturation *in vitro*.

#### PBP2 increases the immunogenic properties of DCs *in vitro* and *in vivo*

**Figure 2:**
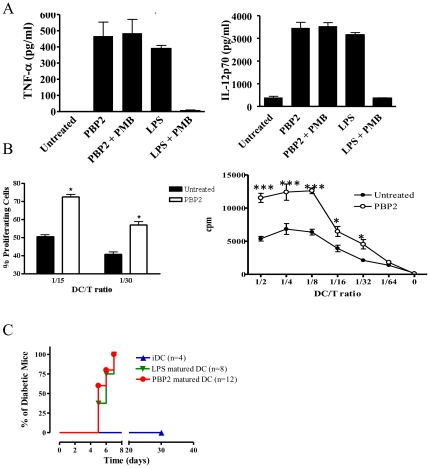
PBP2 increases the immunogenic capacities of DCs. **A:** IL-12p70 and TNF-α levels were quantified in the culture supernatant of DCs cultured in the presence of the indicated compounds for 48 hs. A mean ± SD of four experiments is shown. **B: Left panel:** DDAO-SE-labelled T cells from BALB/c mice were cultured at different ratios for 72 hs with C57/BL6 BMDC previously treated with the indicated compounds. The mean ± SD of divided cells as assessed by DDAO-SE dilution is depicted for two DC/T ratios. n = 2. **Right panel:** Allogeneic untreated and PBP2-treated human monocyte-derived DCs were γ-irradiated and co-cultured for five days with T cells at different DC/T ratios. Proliferation of responder T cells was studied through analysis of ^3^H-thymidine uptake. DCs were washed upon PBP2 treatment minimizing the possibility of a direct effect of PBP2 on T cells. *: p<0.05; **: p<0.01; *: p<0.001. **C:** Diabetes incidence after transfer of untreated (iDC), LPS or PBP2-treated HA-loaded DCs.

Having shown that PBP2 induced DC phenotypic maturation, we then studied whether PBP2 could trigger immunogenic properties in DCs. Before directly addressing this issue, we studied cytokine production by PBP2-treated mouse DCs. In fact, it has been shown that, in addition to increasing expression of MHC class II and co-stimulatory molecules,, fully matured, immunogenic DCs produce important amounts of pro-inflammatory cytokines [15]. Indeed, phenotypically matured DCs failing to produce IL 12p70 have been described to poorly trigger immune responses [15]. We therefore quantified the levels of IL-12p70 as well as TNF-α in the culture supernatant (CSN) of DCs treated with PBP2 using Enzyme linked immunosorbent assay (ELISA) (**Figure 2A**). PBP2 significantly induced IL-12p70 and TNF-α production by mouse DCs as compared to untreated control. Furthermore, LPS-induced IL-12p70 was completely abolished by PMB, yet the treatment did not affect IL-12p70 induction by PBP2, excluding any effect of a contaminant endotoxin (**Figure 2A**). Taken together, our results show that PBP2 triggers DC maturation and that this effect is not due to endotoxin contamination. We therefore addressed whether PBP2 could promote the immunogenic properties of DCs. To study this issue, we first performed *in vitro* allogeneic mixed lymphocyte reactions (MLR). Indeed, the allostimulatory capacity of DCs could be analysed by comparing the proliferation of allogenic T cells upon culture with untreated or PBP2-treated DCs. We studied T cell proliferation by analyzing the dilution of the fluorogenic probe DDAO-SE [10] (**Figure 2B** **left panel**). We observed that the percentage of proliferating allogeneic T cells was increased when stimulatory DCs have been previously treated with PBP2 as compared to untreated cells. PBP2-treated DCs showed similar capacity to stimulate allogeneic T cells as compared to LPS-treated DCs (data not shown). PBP2-treated human monocyte-derived DCs also increased its allostimulatory capacities as shown by 3H-thymidine uptake experiments (**Figure 2B** **right panel**). PBP2 treatment of purified T cells (not co-cultured with DCs) did not induce their proliferation (data not shown). To investigate whether PBP2 treatment promotes immunogenic DC properties *in vivo*, we used a transgenic mouse model in which autoimmune diabetes is induced by mDCs. Ins-HA transgenic mice adoptively transferred with naive anti-HA CD8+ T cells developed diabetes in 6–9 days, only when immunized with both matured and HA peptide-loaded DCs and not when DCs were either immature or not loaded with HA peptide (**Figure 2D**). Importantly, PBP2-treated DC triggered diabetes as effectively as LPS-matured DCs. Altogether, these results show that *in vitro* treatment of mouse DCs with PBP2 increases its immunostimulatory properties *in vitro* and *in vivo*.

### Injection of PBP2 enhances IgG immune response against meningococcal capsule in mice

**Figure 3:**
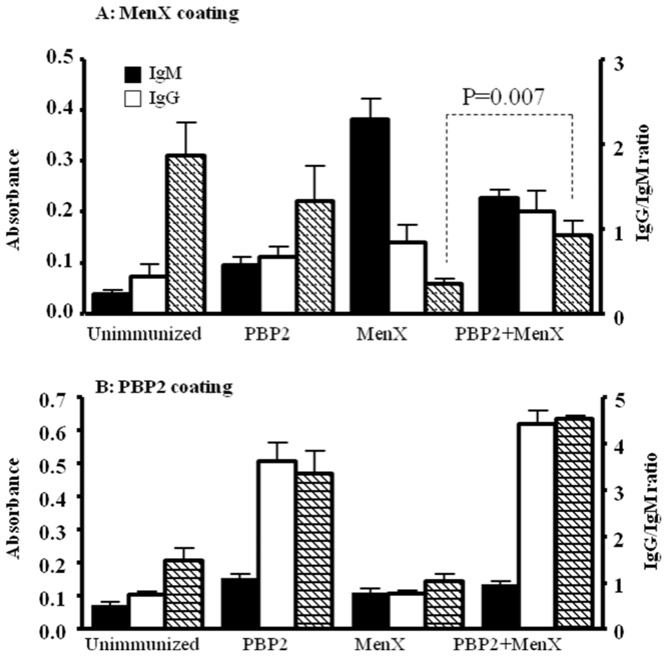
ELISA based screening of IgM and IgG and immune response against purified polysaccharide X and PBP2. ELISA plates were coated with each purified antigen polysaccharide X (A) or PBP2 (B). The tested sera were from unimmunized mice or mice that were immunized with purified polysaccharide X, purified PBP2 or both. Secondary antibodies against the heavy chain of the IgM (Mu chain) and IgG (gamma chain) were used to detect IgM (black boxes) and IgG (white boxes) responses respectively. Data were from three independent experiments with 4 mice in each group. IgG/IgM ratio (hatched boxes) are also shown (right side axis). Significant difference in IgG/IgM ratio is indicated (student *t* test).

**Figure 4:**
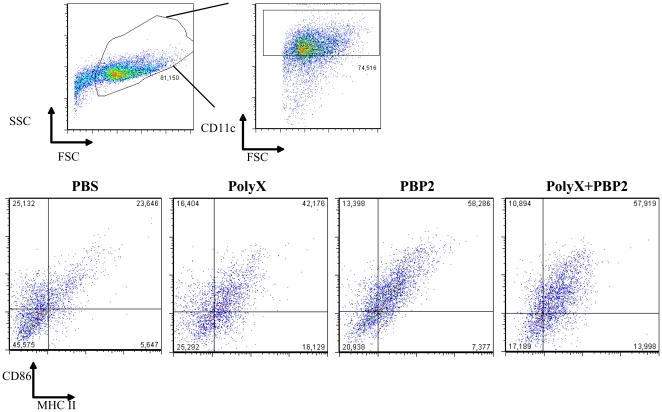
Flow cytometry analysis of dendritic cells extracted from spleens obtained from BALB/c mice immunized with PBP2 or with PBP2+polysaccharide X. The gating strategy used is showed in the upper panel. Stainings were then analyzed in the CD11c^+^ fraction. MHC class II and the co-stimulatory molecule CD86 were analyzed in splenic CD11c^hi^ DCs. The presence of mature DCs under the indicated conditions of immunization was tested 10 days after immunization. CD86 and MHC class II expression was analyzed by FACS. Low levels, if any, of mature DCs was detected in unvaccinated mice and those vaccinated with the polysaccharide alone.

We then explored the ability of PBP2 to work as an immunological adjuvant for anti-meningococcal immune responses. We immunized BALB/c mice subcutaneously with one dose of purified PBP2 (3 µg), or alternatively, with one dose of purified capsular polysaccharide X (20 µg) or both. Mice were sacrificed 10 days later and immune response, IgG production, was determined in blood against both PBP2 and purified capsular polysaccharide of serogroup X by ELISA. As shown in **Figure 3**, the IgG response against polysaccharide was enhanced when both antigens were co-injected in mice while the immune response was mainly IgM when the polysaccharide was used alone to immunize mice. Indeed higher IgG/IgM ratio was observed when both antigens were co-injected compared to the polysaccharide alone. Meningococcal capsular polysaccharide are thymus independent antigens [16]. The response to the protein PBP2 was mainly IgG (Figure 3). These results show that PBP2 can enhance polyclonal immune response against meningococcal antigens *in vivo*. Interestingly, mature DCs were increased in the spleens from immunized mice with PBP2 and both PBP2 and polysaccharide X compared to non immunized or mice immunized with polysaccharide X alone (**Figure 4**). Indeed, our results suggest that PBP2 induces DC maturation *in vivo* promoting immune responses against meningococcal capsule.

### PBP2 induces NF-kB nuclear translocation

**Figure 5:**
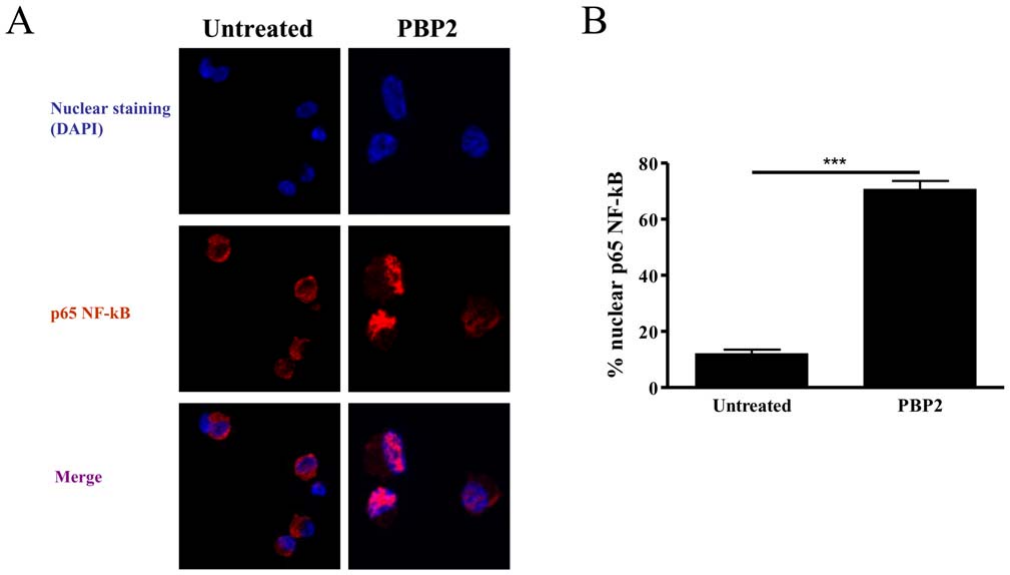
PBP2 induces NF-kB nuclear translocation. Mouse BMDCs were treated with 10 µg/ml of PBP2 or left untreated and 30 minutes later cells were harvested. Subcellular localization of the NF-κB p65 subunit was analyzed using a specific antibody. **A:** representative images depicting anti- NF-κB p65 and nuclear staining. **B:** Quantification of cells showing NF-κB nuclear localization is depicted in the graph. n = 3.

We next aimed to characterize the signalling pathway used by PBP2 to induce DC maturation. We therefore studied whether PBP2 could induce NF-κB activation. In fact, NF-κB activation is a critical event in DC maturation, particularly, in inducing pro-inflammatory cytokines production [17], [18]. To study this issue, we performed confocal microscopy studies analyzing untreated and PBP2-treated DCs which were stained with an anti-p65 NF-κB antibody. We therefore analyzed the subcellular localization (nuclear or cytoplasmic) of p65. Our results clearly showed that PBP2 induced nuclear translocation of p65 NF-κB (**Figure 5A**). A quantification of three different experiments showed that 70.7±5.1% of PBP2-treated DCs showed nuclear localization of p65 NF-κB, whereas only 12±2.6% of untreated DCs displayed that staining pattern (**Figure 5B**). We therefore concluded that PBP2 induces NF- κB nuclear translocation in DCs.

### PBP2-induced DC maturation through TLR4

**Figure 6:**
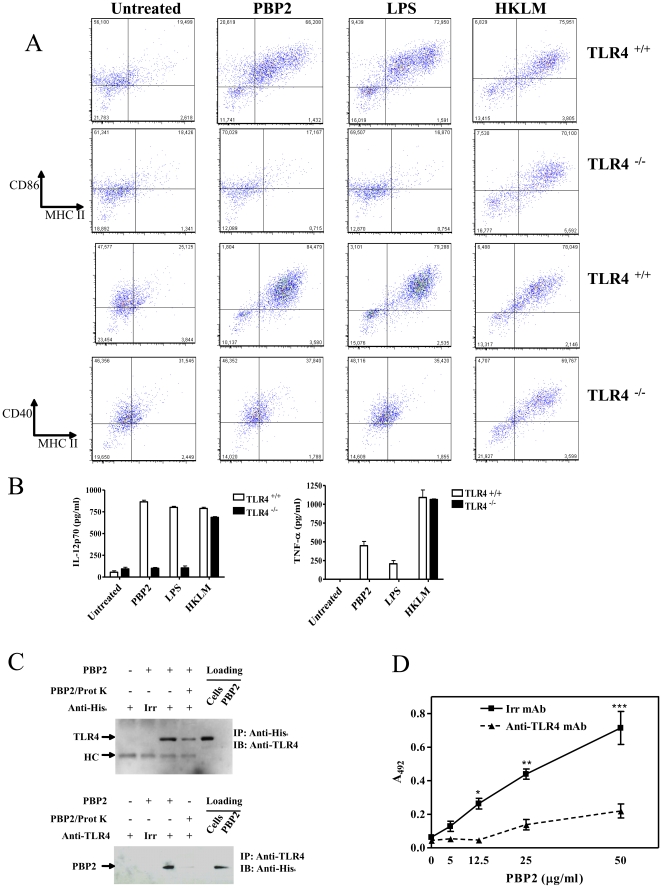
PBP2 fails in inducing maturation in TLR4−/− DCs. WT and TLR4^−/−^ DCs cultured for 48 h in the presence of PBP2 (5 µg/ml), LPS (50 ng/ml) or Heat-killed *L. monocytogenes* (HKLM, 10^8^ CFU/ml). **A:** Analysis of phenotypic markers. One representative experiment of two is shown. **B:** IL-12p70 and TNF-α production in the culture supernatant. The mean ± SD of two independent experiments is shown. **C:** Hec-1-B epithelial cells (expressing TLR4) were treated with 10 µg His6-tagged PBP2. Anti-TLR4 (upper panel) or anti-His6 (lower panel) were used to pull down the target protein. Immunoblot was then performed with anti-His6 (upper panel) or anti-TLR4 (lower panel). **D:** A cellular ELISA assay was performed to show that PBP2 binds to TLR4 on Hec-1-B cells. Cells were treated for 1 h at 37°C with increasing amounts of purified His-tagged PBP2 in presence of anti-TLR4 blocking antibodies (clone HTA125 eBioscience, Hatfield, UK) or an irrelevant normal mouse IgG (10 µg). Bound PBP2 was detected using anti-His-tag polyclonal antibody followed by HRPO-conjugated goat anti-rabbit antibody. In C and D one experiment representative of three is depicted.

NF-κB induction is a common pathway downstream TLRs. We therefore explored the involvement of TLR in mediating the effects of PBP2. Since PBP2 is a bacterial product, TLR2, TLR3 and TLR4 were initially selected because of their involvement in recognition of microbial cell wall components [19], [20], [21]. We therefore studied MHC class II and co-stimulatory molecules expression upon PBP2 treatment of WT and TLR-deficient DCs. No phenotypic differences were found between WT and TLR2^−/−^ and TLR3^−/−^ DCs (**Figure S3**) suggesting that these two TLRs are not involved in PBP2-induced maturation of DCs. In clear contrast, PBP2 as well as LPS were not able to induce maturation of TLR4^−/−^ DCs. It is worth noting that TLR4^−/−^ DCs could be matured when treated with heat-killed *Listeria monocytogenes* (HKLM, TLR2 ligand), showing that TLR4^−/−^ DCs were alive and able to respond to TLR stimulus (**Figure 6A**). These results were concomitant with marked decrease of PBP2-induced IL-12p70 and TNF-α production from TLR4^−/−^ derived DCs compared to WT DCs. (**Figure 6B**). Taken together, these results show that TLR4 is needed for PBP2-induced maturation of DCs. We then studied whether PBP2 could be directly recognized by TLR4. To asses this, we first performed co-immunoprecipitation experiments targeting human TLR4 and PBP2. For these experiments we used Hec-1-B cells, which naturally express TLR4 (data not shown). Hec-1-B epithelial cells were treated with 10 µg of purified His6-tagged PBP2. Remarkably, we observed that PBP2 was co-immunoprecipitated with TLR4 when pull-down was performed with an anti-His tag antibody (**Figure 6C** **, upper panel**) as well as with an anti-TLR4 antibody (**Figure 6C** **, lower panel**). Importantly, through a cellular ELISA assay, we also showed that binding of PBP2 to TLR4-expressing cells was inhibited when adding a blocking anti-TLR4 antibody to the culture (**Figure 6D**). Interestingly, neither the meningococcal regulatory protein CrgA and PBP1 nor PBP2 from *Helicobacter pylori* could interact with TLR4, although are able to induce maturation of DC similarly between WT and TLR4−/− mice (**Figure S4** **and data not shown**). Altogether, our data strongly suggest that meningococcal PBP2 induces DC maturation by directly binding TLR4. Other PBPs may induce DC maturation in TLR4-independent manner.

## Discussion

Host bacteria interaction and output of this interaction is largely dependent on bacterial sensing by host cells [22]. Several bacterial components are now recognized as PAMPs along with their corresponding PRRs [22]. Major surface bacterial components such as lipopolysaccharide and peptidoglycan may be of most importance in host-bacteria interaction owing to their abundance and bioavailability. Other bacterial components may also act as PAMPs. DCs are the most potent antigen presenting cells that initiate and amplify immune responses [23]. Maturation of DCs by PAMPs enables DCs to convey pathogen-associated signals to the adaptive immune system [23]. In this report, we showed that the meningococcal PBP2 can trigger DC maturation through TLR4. PBP2 is a major enzyme involved in the biosynthesis of meningococcal peptidoglycan, most likely by acting as a transpeptidase [1], [24]. Its alteration can modify the structure of peptidoglycan and impact hence on Nod-dependant signalling. It is therefore of interest that PBP2 itself can also directly signal through TLR4. Meningococcal components can hence modulate signalling to host cells [25] and the host's genetic factors can also determine the clinical outcome of meningococcal infection [26]. Interestingly, it has been shown that individuals with rare mutations in TLR4 increase the risk of systemic meningococcal disease [27]. In addition to meningococcal lipooligosaccharide (LOS), PBP2 may also be a relevant and direct player in host-pathogen interactions through TLR4 that influence the clinical outcome of meningococcal infection. Although the canonical TLR4 activator is a liposacharidic structure such as LPS, it is interesting to note that several proteins have been shown to activate this receptor. Indeed, mammalian endogenous proteins such as Tenascin-C [28] and HMGB1 [29] have been described as TLR4 ligands among others. Moreover, F-protein from respiratory syncytial virus (RSV) [30] and FimH adhesion from type 1 fimbriae [31], [32] have been reported to induce DC maturation through TLR4. We directly demonstrate through two different approaches that PBP2 binds human TLR4. This observation opens the exciting perspective of analyzing how this interaction takes place at the molecular level. We observed that neither meningococcal PBP1 nor PBP2 from *H. pylori* could interact with TLR4. Moreover, mutant PBP2 harbouring mutations in the penicillin binding domain, was as effective as WT PBP2 in inducing DC maturation (unpublished observations). We can therefore conclude that the penicillin-binding domain is most likely not involved in TLR4/meningococcal PBP2 interaction.

Our data using several experiment approaches strongly suggest direct interaction between TLR4 and PBP2 that is responsible for the maturation of DC. However, we cannot completely exclude a possible unidentified contaminant in our PBP2 preparation that may be responsible for this effect. This indirect mechanism has been recently suggested to explain the ability to induce cytokine of two intracellular molecular chaperones: the heat shock proteins and the HMGB1 [33].

However, further work is needed to characterize TLR4/PBP2 interaction. For instance, clinically-relevant TLR4 mutations could be analyzed from this perspective. We have previously reported that vaccination with purified recombinant PBP2 was protective against experimental meningococcemia in mice [34]. This protection may be, at least in part, attributed to induction of DC maturation and enhanced T cell proliferation. Further works are needed to explore this possibility. It is noteworthy that PBP2 has a serine-protease domain that may be involved in DC maturation. Interestingly, vaccination with an internal fragment of PBP2a of *Staphylococcus aureus* (comprising the serine-protease domain as antigen) was also reported to induce a protective response against methicillin resistant *Staphylococcus aureus* [35]. PBPs are conserved proteins expressed only on pathogens and not in mammalian cells. Further studies are needed to determine whether other PBPs from meningococci and from other pathogens can work as PAMPs. Meningococcal polysaccharide vaccines have been developed to induce specific antibodies for protection of high-risk populations from meningococcal infections. However, the purified polysaccharide vaccines are less immunogenic, especially in young children -the population in which meningococcal diseases are prevalent-, and is characterized primarily by IgM antibodies, rare IgG and no immunological memory. Conjugation of the polysaccharide to carrier proteins such as tetanus toxoid and diphtheria toxin or its genetic mutant CRM_197_ improved the immunological response in terms of isotype antibody switching from IgM to IgG, which is more protective against meningococcal infection and the anamnestic response [36], [37], [38], [39]. Nevertheless, the use of these proteins as universal carriers may induce a reduction of the response against several vaccines that are administered during infancy and share common protein epitopes by inducing epitopic overload and hapten-specific carrier-induced suppression [40], [41], [42]. The characterization of new carrier proteins seems to be a compulsory step for the achievement of a proper immune response against meningococcal polysaccharide needed for childhood vaccination. Classical adjuvants often contain ligands that stimulate TLR signaling of innate immune cells [43] and vaccines that incorporate ligands for TLR stimulation were shown to boost vaccine responses [44], [45]. PBP2 as a TLR4 ligand may therefore act as an adjuvant through maturation of DCs as also suggested by experiments of co-immunization of PBP2 and capsular polysaccharide with higher IgG immune response and higher IgG/IgM ratio suggesting that PBP2 may have adjuvant properties affecting immunoglobulin class switch for a thymus independent antigen from IgM to IgG [46]. Further studies are needed to determine whether PBP2 is a safer molecule than LPS to be used as an immunological adjuvant *in vivo* [47]. In Summary, our data suggest that PBP2 plays a key role in the interface between innate and adaptive immunity and may represent an interesting candidate to develop anti-meningococcal vaccines or to enhance immune response of other vaccine candidate throughout induction of DC maturation and T-cell proliferation and thus contribute to eliminate the epitopic overload and hapten-specific carrier-induced suppression that could take place when the same protein carrier is used in subsequent vaccination routines.

## Supporting Information

Figure S1 **PBP2 induces DC maturation in a dose-dependent manner.** Mouse BMDC were stimulated for 48 h with the indicated doses of PBP2. The mean fluorescence intensity (MFI) was determined for each maturation marker depicted in the Y axis of the graphics. LOG_EC50_ ± SD calculated from two independent experiments is shown in each graphic (µg/ml).
(PPT)

Figure S2 **Highly purified PBP2 induces DC maturation.** PBP2 issued from Nickel-affinity column has been further purified using anion-exchange chromatography. **A:** FPLC profile of the eluted fractions **B:** SDS-PAGE analysis of PBP2 preparations. *Left panel*, Nickel affinity purified PBP2 (PBP2_NI_ _column_, lane 1) was analyzed along with anion-exchange chromatography eluted fractions F1–F3 (lanes 2–4). The whole cell lysate (WCL) is shown in the left. The gel was submitted to coomassie staining. Only fraction F2 contains PBP2. *Right panel*, decreasing amounts of PBP2 from fraction F2 were further examined for contaminating bands and LPS by silver staining along with decreasing amounts of LPS. Whole cell lysates from bacteria transformed with pET28b empty vector or PBP2-expressing vector pAA2 were shown as controls. Molecular weight markers are indicated. **C:** DCs were left untreated, or alternatively treated with PBP2_NI_ _column_, the eluted fractions F1–F3 or LPS in the presence or not of PMB. DCs were then phenotypically studied by FACS. **D:** PBP2 purified from nickel columns were pre-incubated with anti-PBP2 or irrelevant antibodies and then used to treat DCs which were phenotipically studied by FACS. Numbers represent percentages of each quadrant in FACS figures.
(PPT)

Figure S3 **TLR2 and TLR3 are not needed by PBP2 to induce DC maturation.** Phenotypical analysis of WT, TLR2−/− and TLR3−/− DCs was performed upon PBP2 treatment and compared to untreated cells. Numbers in the dot plots represent the percentage of events in each quadrant.
(PPT)

Figure S4 **Meningococcal CrgA and PBP1 as well as PBP2 from** ***Helicobacter pylori*** **induce DC maturation in a TLR4-independent manner.** **A:** Study of DC phenotypic maturation upon treatment with the indicated proteins in WT and TLR4−/− DCs. The numbers represent the percentage of cells in each quadrant. **B:** Immunoprecipitation studies showing that meningococcal PBP2 but not the other proteins studied co immunoprecipitates with TLR4.
(PPT)
