# Supplementary material for: Penicillin Binding Proteins as Danger Signals: Meningococcal Penicillin Binding Protein 2 Activates Dendritic Cells through Toll-Like Receptor 4
Source: PLoS One. 2011 Oct 27;6(10):e23995. doi: 10.1371/journal.pone.0023995 (PMC3203111; doi:10.1371/journal.pone.0023995)
Supplement: Figure S1 — PBP2 induces DC maturation in a dose-dependent manner. Mouse BMDC were stimulated for 48 h with the indicated doses of PBP2. The mean fluorescence intensity (MFI) was determined for each maturation marker depicted in the Y axis of the graphics. LOGEC50 ± SD calculated from two independent experiments is shown in each graphic (µg/ml). (PPT) [file pone.0023995.s001.ppt]

## Slide 1
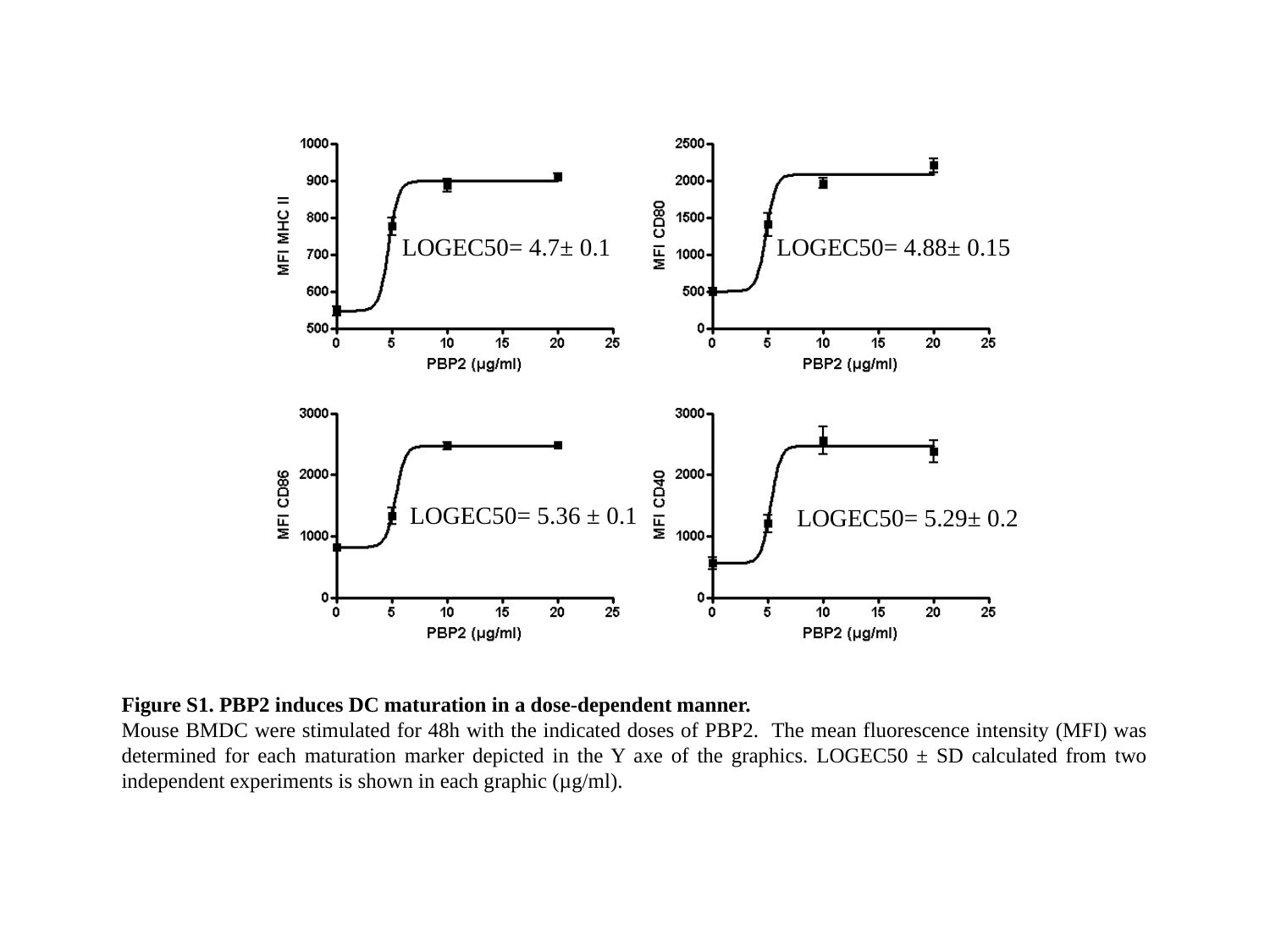

LOGEC50= 4.7± 0.1
LOGEC50= 4.88± 0.15
LOGEC50= 5.36 ± 0.1
LOGEC50= 5.29± 0.2
Figure S1. PBP2 induces DC maturation in a dose-dependent manner.
Mouse BMDC were stimulated for 48h with the indicated doses of PBP2. The mean fluorescence intensity (MFI) was determined for each maturation marker depicted in the Y axe of the graphics. LOGEC50 ± SD calculated from two independent experiments is shown in each graphic (µg/ml).
