# Supplementary material for: Penicillin Binding Proteins as Danger Signals: Meningococcal Penicillin Binding Protein 2 Activates Dendritic Cells through Toll-Like Receptor 4
Source: PLoS One. 2011 Oct 27;6(10):e23995. doi: 10.1371/journal.pone.0023995 (PMC3203111; doi:10.1371/journal.pone.0023995)
Supplement: Figure S2 — Highly purified PBP2 induces DC maturation. PBP2 issued from Nickel-affinity column has been further purified using anion-exchange chromatography. A: FPLC profile of the eluted fractions B: SDS-PAGE analysis of PBP2 preparations. Left panel, Nickel affinity purified PBP2 (PBP2NI column, lane 1) was analyzed along with anion-exchange chromatography eluted fractions F1–F3 (lanes 2–4). The whole cell lysate (WCL) is shown in the left. The gel was submitted to coomassie staining. Only fraction F2 contains PBP2. Right panel, decreasing amounts of PBP2 from fraction F2 were further examined for contaminating bands and LPS by silver staining along with decreasing amounts of LPS. Whole cell lysates from bacteria transformed with pET28b empty vector or PBP2-expressing vector pAA2 were shown as controls. Molecular weight markers are indicated. C: DCs were left untreated, or alternatively treated with PBP2NI column, the eluted fractions F1–F3 or LPS in the presence or not of PMB. DCs were then phenotypically studied by FACS. D: PBP2 purified from nickel columns were pre-incubated with anti-PBP2 or irrelevant antibodies and then used to treat DCs which were phenotipically studied by FACS. Numbers represent percentages of each quadrant in FACS figures. (PPT) [file pone.0023995.s002.ppt]

## Slide 1
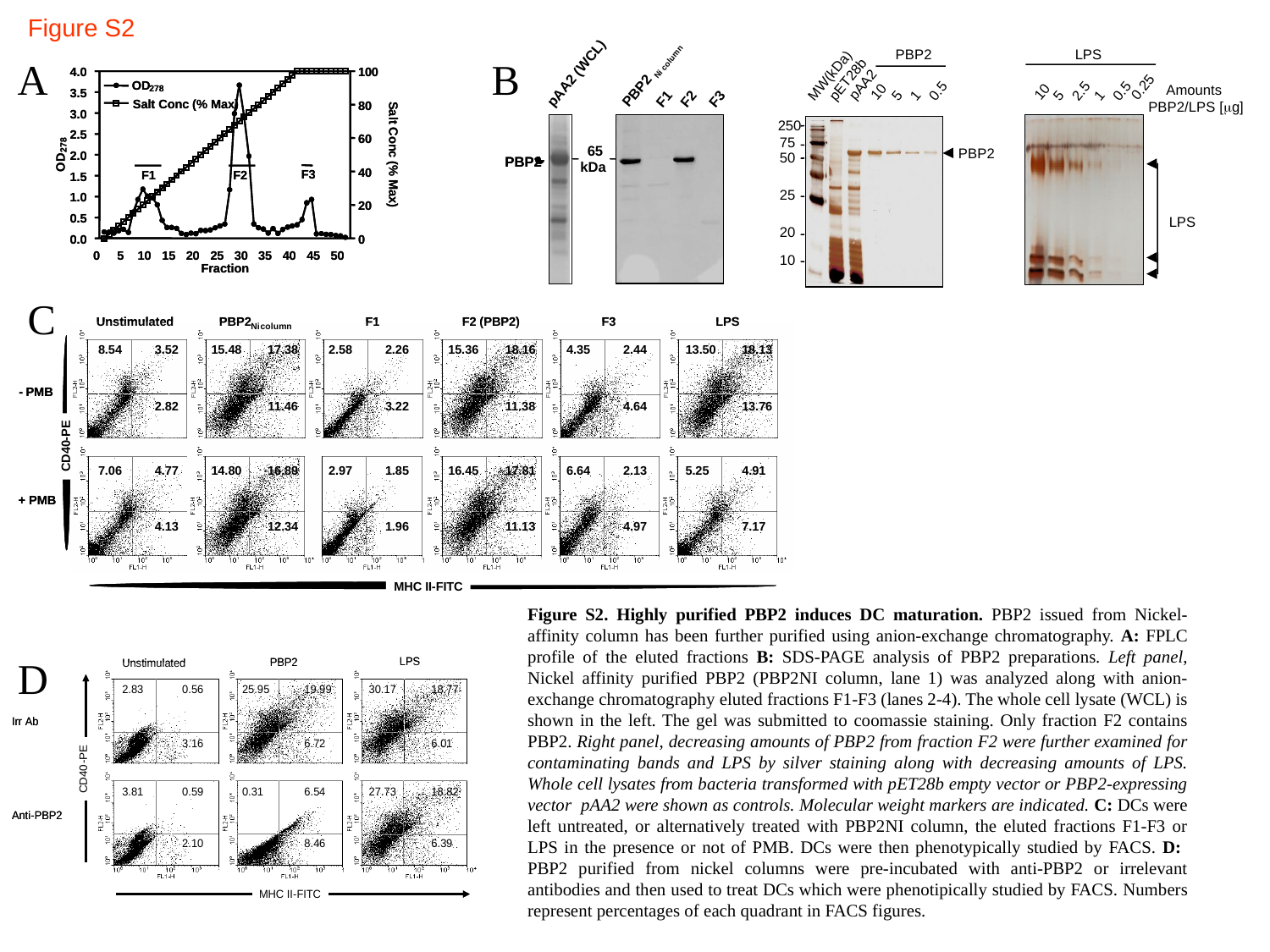

Figure S2
PBP2
LPS
0.25
Amounts
PBP2/LPS [μg]
2.5
0.5
10
5
1
LPS
MW(kDa)
pET28b
pAA2
0.5
10
1
5
250
75
PBP2
50
25
20
10
pAA2 (WCL)
PBP2 Ni column
F2
F3
F1
65
kDa
PBP2
PBP2
B
A
4.0
100
OD
278
3.5
Salt Conc (% Max)
80
3.0
2.5
60
278
Salt Conc (% Max)
2.0
OD
40
1.5
1.0
20
0.5
0.0
0
0
5
10
15
20
25
30
35
40
45
50
Fraction
4.0
100
OD
278
3.5
Salt Conc (% Max)
80
3.0
2.5
60
278
Salt Conc (% Max)
2.0
OD
40
1.5
1.0
20
0.5
0.0
0
0
5
10
15
20
25
30
35
40
45
50
Fraction
F3
F3
F2
F2
F1
F1
C
Unstimulated
Unstimulated
PBP2
PBP2
F1
F1
F2 (PBP2)
F2 (PBP2)
F3
F3
LPS
LPS
Ni
Ni
column
column
8.54
8.54
3.52
3.52
15.48
15.48
17.38
17.38
2.58
2.58
2.26
2.26
15.36
15.36
18.16
18.16
4.35
4.35
2.44
2.44
13.50
13.50
18.13
18.13
-
-
PMB
PMB
2.82
2.82
11.46
11.46
3.22
3.22
11.38
11.38
4.64
4.64
13.76
13.76
PE
PE
-
-
CD40
CD40
7.06
7.06
4.77
4.77
14.80
14.80
16.89
16.89
2.97
2.97
1.85
1.85
16.45
16.45
17.81
17.81
6.64
6.64
2.13
2.13
5.25
5.25
4.91
4.91
+ PMB
+ PMB
4.13
4.13
12.34
12.34
1.96
1.96
11.13
11.13
4.97
4.97
7.17
7.17
MHC II
MHC II
-
-
FITC
FITC
Figure S2. Highly purified PBP2 induces DC maturation. PBP2 issued from Nickel-affinity column has been further purified using anion-exchange chromatography. A: FPLC profile of the eluted fractions B: SDS-PAGE analysis of PBP2 preparations. Left panel, Nickel affinity purified PBP2 (PBP2NI column, lane 1) was analyzed along with anion-exchange chromatography eluted fractions F1-F3 (lanes 2-4). The whole cell lysate (WCL) is shown in the left. The gel was submitted to coomassie staining. Only fraction F2 contains PBP2. Right panel, decreasing amounts of PBP2 from fraction F2 were further examined for contaminating bands and LPS by silver staining along with decreasing amounts of LPS. Whole cell lysates from bacteria transformed with pET28b empty vector or PBP2-expressing vector pAA2 were shown as controls. Molecular weight markers are indicated. C: DCs were left untreated, or alternatively treated with PBP2NI column, the eluted fractions F1-F3 or LPS in the presence or not of PMB. DCs were then phenotypically studied by FACS. D: PBP2 purified from nickel columns were pre-incubated with anti-PBP2 or irrelevant antibodies and then used to treat DCs which were phenotipically studied by FACS. Numbers represent percentages of each quadrant in FACS figures.
D
