# Supplementary material for: Penicillin Binding Proteins as Danger Signals: Meningococcal Penicillin Binding Protein 2 Activates Dendritic Cells through Toll-Like Receptor 4
Source: PLoS One. 2011 Oct 27;6(10):e23995. doi: 10.1371/journal.pone.0023995 (PMC3203111; doi:10.1371/journal.pone.0023995)
Supplement: Figure S3 — TLR2 and TLR3 are not needed by PBP2 to induce DC maturation. Phenotypical analysis of WT, TLR2−/− and TLR3−/− DCs was performed upon PBP2 treatment and compared to untreated cells. Numbers in the dot plots represent the percentage of events in each quadrant. (PPT) [file pone.0023995.s003.ppt]

## Slide 1
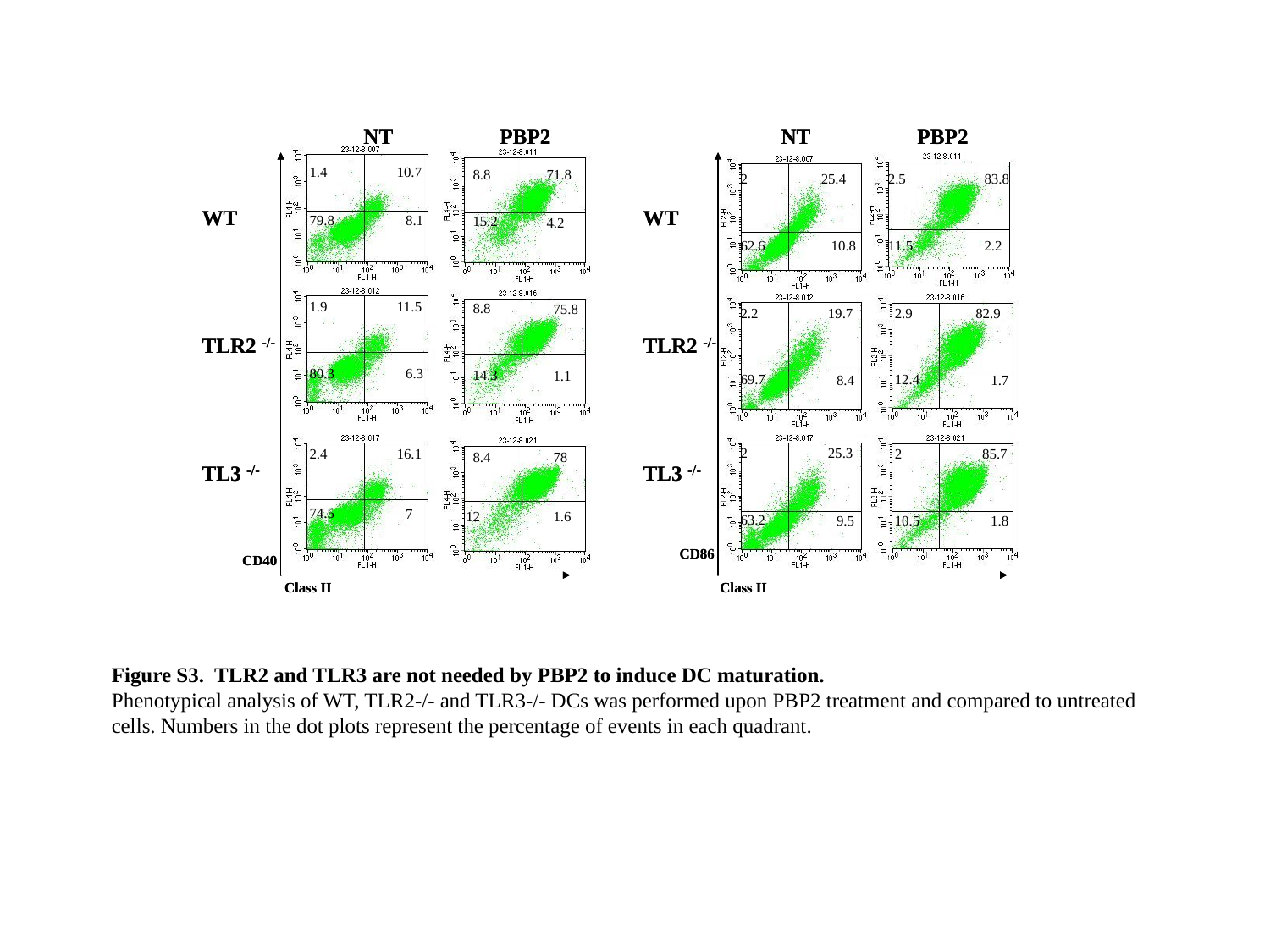

Figure S3. TLR2 and TLR3 are not needed by PBP2 to induce DC maturation.
Phenotypical analysis of WT, TLR2-/- and TLR3-/- DCs was performed upon PBP2 treatment and compared to untreated cells. Numbers in the dot plots represent the percentage of events in each quadrant.
