# Supplementary material for: Penicillin Binding Proteins as Danger Signals: Meningococcal Penicillin Binding Protein 2 Activates Dendritic Cells through Toll-Like Receptor 4
Source: PLoS One. 2011 Oct 27;6(10):e23995. doi: 10.1371/journal.pone.0023995 (PMC3203111; doi:10.1371/journal.pone.0023995)
Supplement: Figure S4 — Meningococcal CrgA and PBP1 as well as PBP2 from Helicobacter pylori induce DC maturation in a TLR4-independent manner. A: Study of DC phenotypic maturation upon treatment with the indicated proteins in WT and TLR4−/− DCs. The numbers represent the percentage of cells in each quadrant. B: Immunoprecipitation studies showing that meningococcal PBP2 but not the other proteins studied co immunoprecipitates with TLR4. (PPT) [file pone.0023995.s004.ppt]

## Slide 1
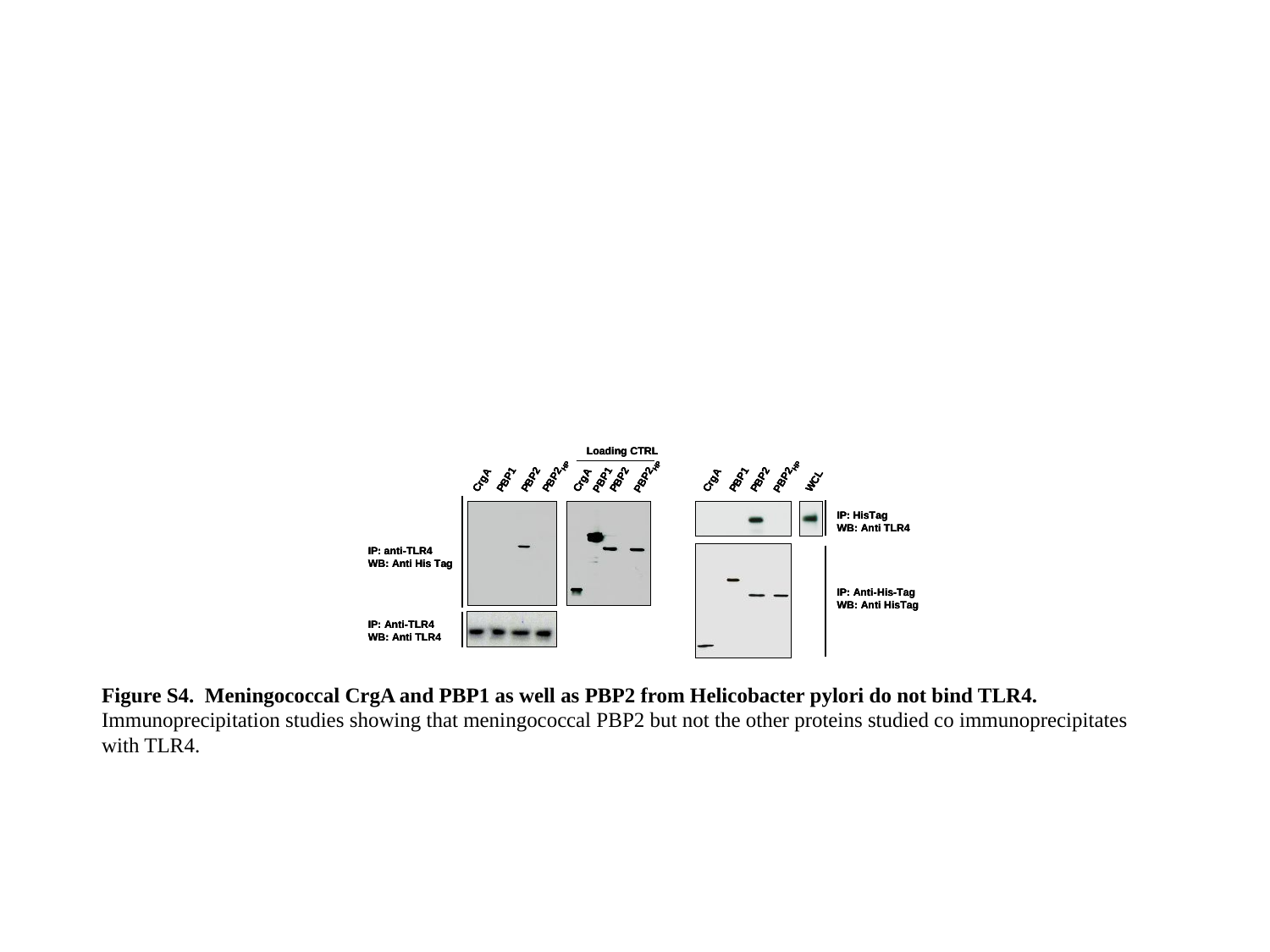

Figure S4. Meningococcal CrgA and PBP1 as well as PBP2 from Helicobacter pylori do not bind TLR4.
Immunoprecipitation studies showing that meningococcal PBP2 but not the other proteins studied co immunoprecipitates with TLR4.
